# Formulation and Statistical Optimization of Culture Medium for Improved Production of Antimicrobial Compound by *Streptomyces* sp. JAJ06

**DOI:** 10.1155/2013/526260

**Published:** 2013-12-23

**Authors:** Polpass Arul Jose, Kunjukrishnan Kamalakshi Sivakala, Solomon Robinson David Jebakumar

**Affiliations:** Department of Molecular Microbiology, School of Biotechnology, Madurai Kamaraj University, Madurai 625 021, India

## Abstract

*Streptomyces* sp. JAJ06 is a seawater-dependent antibiotic producer, previously isolated and characterised from an Indian coastal solar saltern. This paper reports replacement of seawater with a defined salt formulation in production medium and subsequent statistical media optimization to ensure consistent as well as improved antibiotic production by *Streptomyces* sp. JAJ06. This strain was observed to be proficient to produce antibiotic compound with incorporation of chemically defined sodium-chloride-based salt formulation instead of seawater into the production medium. Plackett-Burman design experiment was applied, and three media constituents, starch, KBr, and CaCO_3_, were recognised to have significant effect on the antibiotic production of *Streptomyces* JAJ06 at their individual levels. Subsequently, Response surface methodology with Box-Behnken design was employed to optimize these influencing medium constituents for the improved antibiotic production of *Streptomyces* sp. JAJ06. A total of 17 experiments were conducted towards the construction of a quadratic model and a second-order polynomial equation. Optimum levels of medium constituents were obtained by analysis of the model and numerical optimization method. When the strain JAJ06 was cultivated in the optimized medium, the antibiotic activity was increased to 173.3 U/mL, 26.8% increase as compared to the original (136.7 U/mL). This study found a useful way to cultivate *Streptomyces* sp. JAJ06 for enhanced production of antibiotic compound.

## 1. Introduction

*Streptomyces* are the prime producers of bioactive compounds for biotechnology industry. Several clinically significant antibiotics as well as widely used drugs against common diseases have been derived from this unique genus affiliated with the order Actinomycetales [1]. Diverse members of this order including *Streptomyces* have been isolated from previously unexplored natural habitats to nourish the current microbial antibiotic searching programs [2, 3]. *Streptomyces *sp. JAJ06 is a Gram-positive, moderately halophilic *Streptomyces *strain, which has previously been isolated from hypersaline coastal solar saltern at Tuticorin, India [4]. This strain produces an antimicrobial polyketide compound which has been reported to have potent antimicrobial activity against a set of bacteria and yeast with significant minimal inhibitory concentrations [5]. Production of antibiotic compound in JAJ06 has been reported to have seawater dependence. Like many other complex medium components, seawater has nondefined composition which might affect the reproducibility of the production profile of microorganisms [6]. To maintain a reproducible production profile, defined salt formulations can be used.

Media components and their optimum levels are critical to the secondary metabolites produced by microorganisms. In the field of antibiotics, much effort was directed toward optimizing production rates and directing the product spectrum [7]. Production of microbial secondary metabolites in a microbial system can be improved by optimization of physical parameters and nutritional constituents of production medium [8]. The optimization experiments are usually performed using nonstatistical one-factor-at-a-time [9, 10] and statistical experimental design approaches [11, 12]. However, the former one is highly laborious and time consuming than the statistical methods [13]. Consequently, statistical experimental design techniques especially Plackett-Burman design (PBD) and response surface methodology (RSM) are widely used to select the significant variables and obtain the optimal levels, respectively [7, 11–18]. The application of these statistical experimental design techniques in media optimization can result in improved product yields, reduced process variability, reduced time, and overall costs, compared with conventional practice of single factor optimization [7, 13]. Plackett-Burman design has been applied by several researchers to select influencing factors among the constituents of complex medium [15–17]. Optimization of selected, highly influencing factors can be done using response surface methodology with either central composite design (CCD) [7] or Box-Behnken Design experiments [18].

In present work, efforts taken to expel the seawater from the production medium of JAJ06, with incorporation of chemically defined salt formulations. Further, in order to enhance the antibiotic production by JAJ06, production medium was optimized using a successive optimization strategy, selection of media components that significantly influence the antibiotic production using Plackett-Burman design and optimization of these media components using RSM with Box-Behnken design.

## 2. Materials and Methods

### 2.1. Strain and Culture Condition

The antibacterial compound producing strain, *Streptomyces* sp. JAJ06 was previously isolated from a coastal solar saltern soil and extensively studied for their antibacterial secondary metabolite [5]. The strain was maintained over the surface of ISP4 agar slants supplemented with 3% of sea salt (w/v).

### 2.2. Basal Production Medium and Salt Formulation

A nutrient medium contained 10 g of starch, 4 g of yeast extract, 2 g of peptone, 1 g of MgSO_4_, 2 g of CaCO_3,_ 0.04 g of FeSO_4_·7H_2_O, and 0.1 g of KBr in 1 L of sterile seawater was taken as basal production medium for further modification and statistical optimization.

In order to replace the chemically nondefined seawater, two defined salt formulations were screened and compared with seawater to support antibiotic compound in JAJ06. According to previous reports, two salt formulations were prepared with some modifications: salt formulation I [6] contained 12 g of NaCl, 0.35 g of KCl, 0.22 g of CaCl_2_·2H_2_O, 10.7 mg of H_3_BO_3_, 7.3 mg of SrCl_2_, 1.3 mg of NaF, and 26 *μ*g of CoCl_2_·6H_2_O in 1 liter of deionized water; salt formulation II [19] contained 15 g of KCl, 0.22 g of CaCl_2_·2H_2_O, 10.7 mg of H_3_BO_3_, 7.5 mg of SrCl_2_, 1.3 mg of NaF, and 26 *μ*g of CoCl_2_·6H_2_O in 1 liter of deionized water.

### 2.3. Seed Stock and Culturing Conditions

Spore suspension of JAJ06 was prepared in distilled water from cultures grown on modified ISP-4 medium supplemented with 3% of sea salt (w/v) at 30°C for 7 days. The suspension was then added to ISP-2 broth in 250 mL Erlenmeyer flask at a rate of 10^8^ spores in 50 mL liquid medium. Cultures were incubated on a shaker at 120 rpm at 30°C for 3 days and used as seed stocks. For antibiotic production, strain JAJ06 was inoculated into production medium and incubated on a shaker at 120 rpm for 8 days at 30°C.

### 2.4. Extraction of Antibiotic Substance

Cell-free supernatant of fermentation broth was recovered by centrifuging it at 10000 rpm for 10 min. Ethyl acetate was added to the supernatant in 1 : 1 proportion and the mixture was agitated for 10 min. The solvent layer containing antibiotic substance was separated from broth and it was further centrifuged at 5000 rpm for 15 min to remove traces of fermentation broth. The antimicrobial crude extract was concentrated tenfold using a rotational vacuum concentrator and used for antibacterial assay.

### 2.5. Antimicrobial Assay

The extracted crude substance was assayed in triplicate for their antimicrobial activity against *Bacillus subtilis* MTCC 441 by agar diffusion plate assay [7, 20]. The crude extract was loaded on 6 mm sterile discs, dried, and placed on nutrient agar plate inoculated with *B. subtilis* suspension adjusted to a McFarland standard of 0.5, which is equivalent to 1.5 × 10^8^ CFU/mL. A sterile disc impregnated with ethyl acetate was used as control. The plates were incubated at 37°C for 24 h and the inhibition zone formed around the disc was measured with transparent ruler in millimeter. Maxwell et al. [20] and Wang et al. [7] confirmed that the size of the zones of inhibition can be considered as measure of antibiotic titre. Therefore, the antibiotic activity was expressed as units of activity per millilitre the crude substance of culture, where 1 U was defined as a 1.0 mm annular clearing around the antibiotic disk.

### 2.6. Screening for Essential Medium Components Using Plackett-Burman Design

Plackett-Burman design (PBD) was employed for screening the most significant medium components for growth and antimicrobial compound production by *Streptomyces *sp. JAJ06. Minitab 15.0 (Minitab Inc., PA, USA) was used for the experimental designs and subsequent analysis of the experimental data. In the experimental design, 7 medium components (independent variables) were screened by representing them at two levels, low (−) and high (+) in 12 trials.  Table 1 shows media components, symbol code, and actual low and high level of the variables.  Table 2 shows the detail of the design, each row represents a trial, and each column represents an independent variable. The experiment was carried out in triplicate and the average antimicrobial activity against *B. subtitles* was taken as the response. The variables with confidence levels above 90% were considered to have significant effect on antimicrobial compound production and thus used for further optimization.

### 2.7. Optimization of Selected Ingredients by RSM

Response surface methodology (RSM) was used with Box-Behnken design to optimize the selected media constituents: starch, KBr, and CaCO_3_ for enhanced antibiotic production in JAJ06. The three medium components (independent variables) were studied at three different levels: (−), (0), and (+) for low, intermediate, and high concentrations, respectively (Table 3). The experiment was carried out in 17 trials (Table 4) with five replicates at the centre point and the values of responses were the mean of two replications. For statistical calculations, coding of the factors was done according to the following equation:

(1) $$\begin{array}{c} x_{i}=\frac{X_{i}-X_{0}}{\delta X_{i}},\;i=0,1,2,3,\ldots ,n, \end{array}$$

where *x*_*i*_ was the coded value of an independent factor, *X*_*i*_ was the actual value of an independent factor, *X*_0_ was the actual value of an independent factor at the center point, and *δX*_*i*_ was the step change value.

For predicting the optimal point, a second-order model was fitted to correlate the relationship between independent variables and response. The behaviour of the system was explained by the following quadratic equation:

(2) $$\begin{array}{c} Y=\beta_{0}+\Sigma \beta_{i}X_{i}+\Sigma \beta_{ij}X_{i}X_{j}+\Sigma \beta_{ii}X_{i}^{2}, \end{array}$$

where *Y* is the predicted response, *β*_0_ is the intercept term, *β*_*i*_ is the linear coefficient, *β*_*ij*_ is the quadratic coefficient, *β*_*ii*_ is the interaction coefficient, and *X*_*i*_ *X*_*j*_ represent the independent variables.

The Design Expert trial package (Version 7.0) was used for the experimental design and the regression analysis of the data obtained. The statistical significance of the model was verified by applying the analysis of variance (ANOVA). Overall model significance was determined using Fisher's *F*-test and its associated probability *P*(*F*). The lack of fit was also applied to estimate the model. Lack of fit values lower than 0.05 indicates that there might be a contribution to the variables-response relationship that the model does not take into account. The quality of the polynomial model equation was judged statistically by coefficient of determination (*R* ^2^) and adjusted *R* ^2^. The fitted polynomial equation was then expressed in the form of three-dimensional response surface plots, to illustrate the relationship between the responses and the experimental levels of each independent variable. The Design Expert's numerical optimization method was employed to optimize the level of each variable for maximum response.

### 2.8. Experimental Validation

The combination of different optimized variables, which yielded the maximum response, was experimentally validated by culturing JAJ06 in optimized and unoptimized production medium. The cell-free culture broths were collected and extracted with equal volume of ethyl acetate and the top organic layer was dried for further analysis. The dried ethyl acetate extracts were resuspended in methanol and assayed as above for antibiotic activity.

## 3. Results

### 3.1. Chemically Defined Salt Formulation for JAJ06

The maximal antibiotic activity of JAJ06 in three different media prepared separately with nondefined sterile seawater and two chemically defined salt formulations was examined in shake flask cultures. Growth and antibiotic activity exerted by *Streptomyces* sp. JAJ06 in production media containing various salt compositions are given in Table 5. The sodium chloride based salt formulation I supported a better antibiotic activity than seawater. However growth rate in both seawater and sodium chloride based salt formulation seemed to be the same, while the growth rate and antibiotic activity are slightly lower in salt formulation II than those of the other two.

### 3.2. Screening for Essential Medium Components Using PB Design

**Figure 1:**
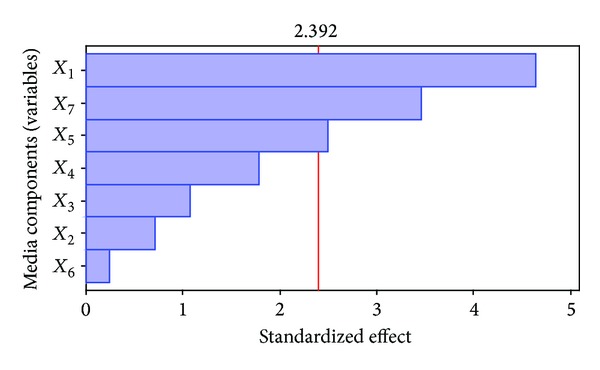
Pareto chart showing the effect of different media components (variables) on antibiotic activity based on the observations of Plackett-Burman design.

The main effect of each variable on antibiotic activity was calculated to determine the medium components which influence the antimicrobial compound production by *Streptomyces *sp. JAJ06.  Table 6 represents the effect, standard error, *t*-value, *P* value, and confidence level of each component from the result of antibiotic assay given in Table 2. The medium components were screened at the confidence level of 92.5% on the basis of their effects. It was indicated that starch (*X*_1_), KBr (*X*_5_), and CaCO_3_ (*X*_7_) had apparent influences in antibiotic production by JAJ06. Confidence levels of these three variables were higher than 90% which indicates their significant contributions than those of other media components. The same was confirmed from the Pareto graph (Figure 1) in which higher effects were presented in the upper portion and then progress down to the lower effects. It directly shows that the most important factors influencing antimicrobial compound production were starch, KBr, and CaCO_3_.

### 3.3. Optimization of Selected Medium Components

Based on the results of the Plackett-Burman design, starch, KBr, and CaCO_3_ were chosen as most influencing media components and further optimised using response surface methodology. In this approach, the batch runs were conducted in Box-Behnken design experiments and results to determine the effect of independent factors on the response along with the predicted values are shown in Table 4. The regression equation coefficients were calculated and the data was fitted to a second-order polynomial equation. The response of antibiotic activity (*Y*) can be expressed in terms of the following regression equation:

(3) Y  (Antibiotic  activity) =165.34+13.54A+7.29B  −5.83C−0.43AB+2.50AC+0.85BC  −14.13A2−21.63B2−18.71C2,

where *Y* represented the response of antibiotic activity, and *A*, *B*, and *C* were the coded values of starch, KBr, and CaCO_3_, respectively.

The ANOVA was performed to inspect the second-order response surface model and results are given in Table 7. The ANOVA showed that the model was highly significant, as it was evident from the low *P* value (<0.0001) of the Fisher's *F*-test. Significance of model was also supported by statistically insignificant lack of fit, as was evident from the lower calculated *F* value (4.01). The model *R* ^2^ of 0.9831 implied that model equation could explain 98.31% of the total variation which suggested a good agreement between predicated values and experimental data.

**Figure 2:**
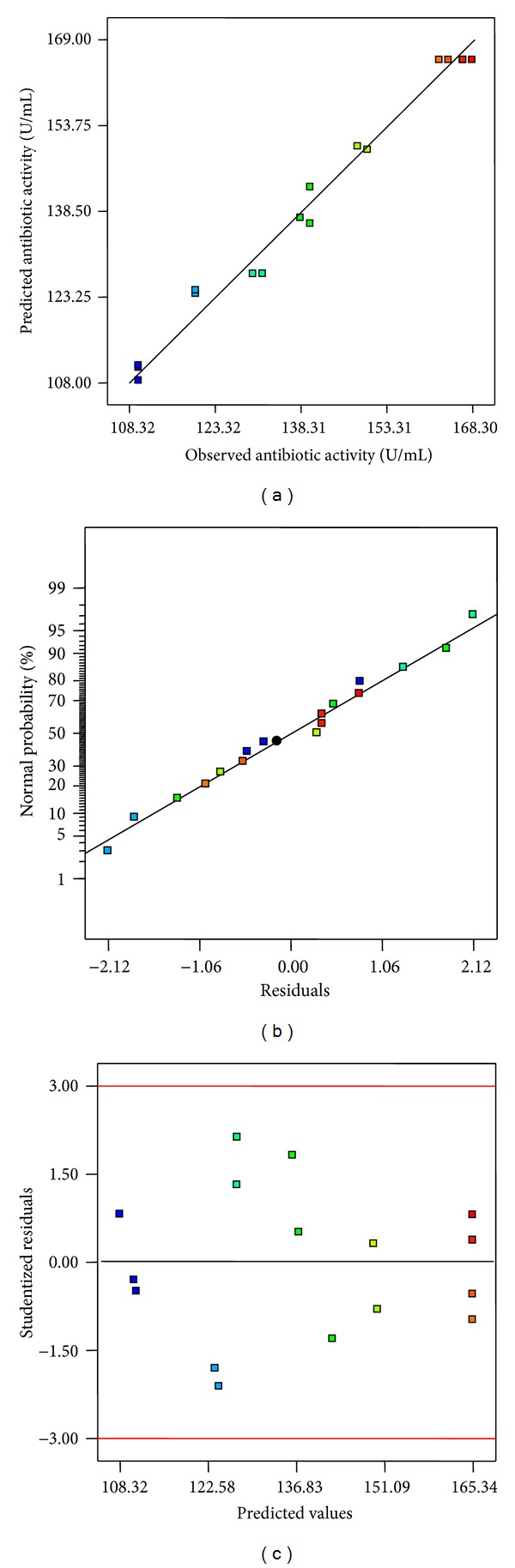
Residual diagnostic plots of quadratic model. (a) Observed versus predicted response plot. (b) Normal probability plot of the studentized residuals. (c) Internally studentized residuals versus predicted response plot.

Diagnostic plots were drawn to judge the model adequacy and clarify the signs of any problems in the experimental data. Plot of observed response (antibiotic activity) versus predicted reponse is shown in Figure 2(a). In this case, predicted values were in agreement with observed ones in the range of the operating variables. The normal probability plot of the studentized residuals was used to check for normality of residuals (Figure 2(b)). A linear pattern observed in this plot suggests that there was no sign of any problem in the experimental data.  Figure 2(c) represents a plot of studentized residuals versus predicted values to check for constant error. Residuals displayed randomness in scattering and suggested that the variance of the original observation was constant.

**Figure 3:**
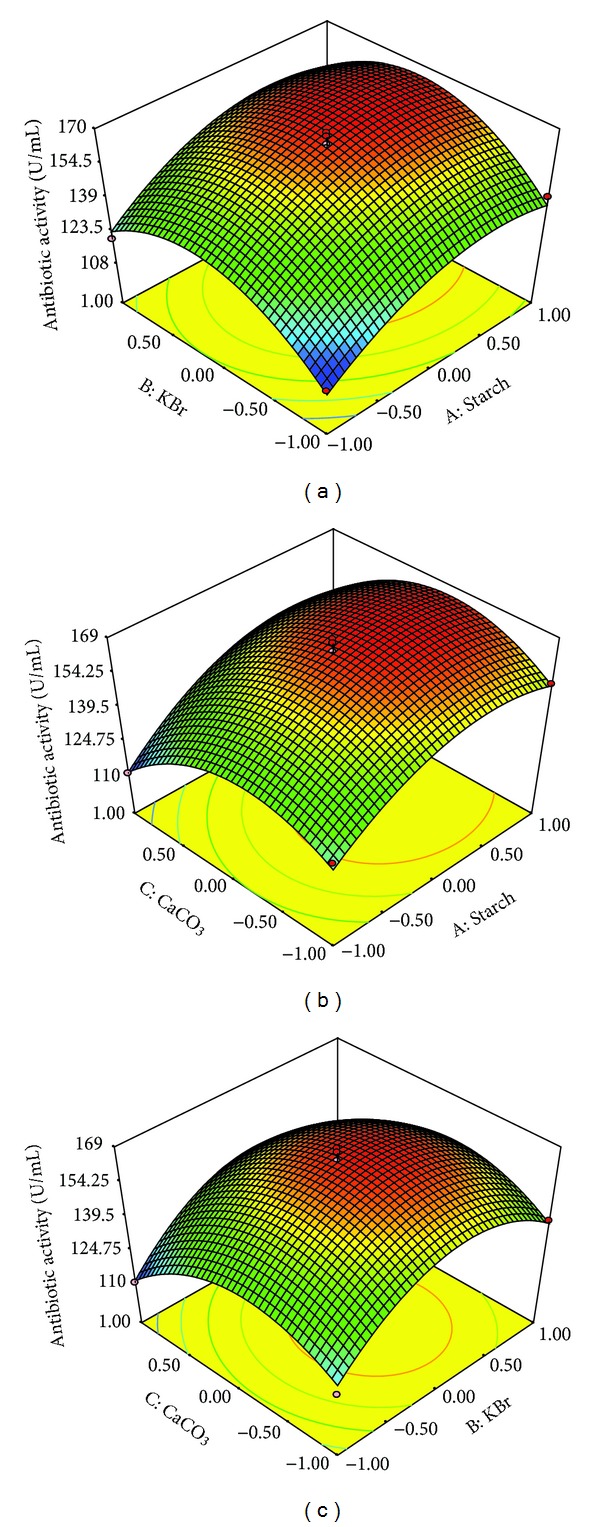
Response surface plots showing individual and interactive effects of variables on antibiotic activity of *Streptomyces* sp. JAJ06. (a) Effects of starch and KBr on antibiotic activity. (b) Effects of starch and CaCO_3_ on antibiotic activity. (c) Effects of KBr and CaCO_3_ on antibiotic activity.

The three-dimensional (3D) response surface plots were drawn to illustrate the individual and interactive effects of starch, KBr, and CaCO_3_ on antibiotic production by JAJ06 (Figure 3). Each 3D plot presented the effects of two variables while the rest one was held at middle level. There was insignificant mutual interaction between starch and KBr as well as starch and CaCO_3_ (Figures 3(a) and 3(b)), respectively. With the increase of the concentration of starch from 2 to 17.5 g/L (coded value, −1.0 to 0.75), the antibiotic activity significantly increased at moderate concentration of KBr (coded value, 0.5); however, decreased antibiotic activity was observed with any further increase of KBr even at high concentration of starch (Figure 3(a)).

When the concentration of CaCO_3_ was just beyond the middle level (coded value, 0.25), the antibiotic activity increased with increasing concentration of starch from 2 to 15 g/L (coded value, −0.1 to 0.5) and further increase of starch to its high level resulted mild decrease in antibiotic production (Figure 3(b)).

With the increase of the concentration of KBr from 0.02 to 1.1 g/L (coded value, −1 to 0.15), the antibiotic activity significantly increased to certain level at a low concentration of CaCO_3_ (coded value, −1) and further slightly increased at a moderate level of CaCO_3_ (coded value, 0.25). Lower concentration of CaCO_3_ was found to be supportive for antibiotic activity; however the activity was suppressed when the concentration of CaCO_3_ was higher in production medium.

### 3.4. Optimization and Experimental Validation

**Figure 4:**
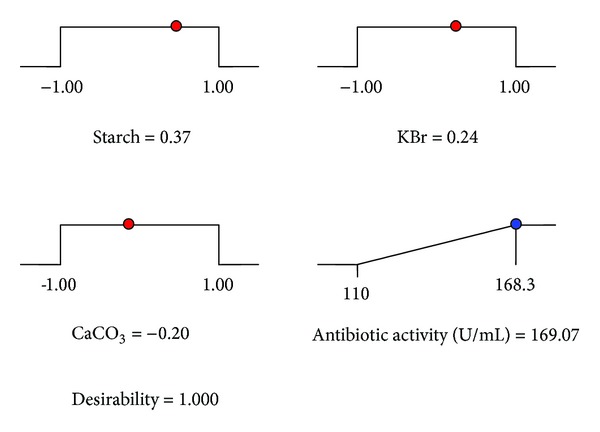
Summary of criteria set for optimization run. Ramps show the predicted levels of variables in the range of given concentration (coded values) and predicted possible antibiotic activity.

On the basis of numerical optimization, the quadratic model predicted that the maximum antibiotic activity was 169.07 U/mL, when the optimal values of test factors in the coded units were starch = 0.37, KBr = 0.24, and CaCO_3_ = −0.20 (Figure 4), which were 7.4 g/L starch, 0.048 g/L KBr, and 0.8 g/L CaCO_3_, respectively. The validation of the statistical results using the optimized medium was accomplished by carrying out shake-flask experiments in triplicate. The maximum antibiotic activity unit obtained experimentally was found to be 173.3 U/mL, (Table 8) which is in close agreement with the prediction value (169.07 U/mL). Therefore, the developed model was considered to be accurate and reliable for predicting the production of antibiotic by *Streptomyces* sp. JAJ06. The final optimized medium contained 7.4 g of starch, 4 g of yeast extract, 2 g of peptone, 1 g of MgSO_4_, 0.8 g of CaCO_3,_ 0.04 g of FeSO_4_·7H_2_O, and 0.048 g of KBr in 1 L of sodium chloride based salt formulation I.

## 4. Discussion

*Streptomyces* are potential producers of secondary metabolites with antibiotic properties [21–23]. Their antibiotic producing capability is not a static property and it can be significantly affected by constituents of production medium [24, 25]. Present investigation focused primarily on improvement of antibiotic production by *Streptomyces* sp. JAJ06 as a function of levels of constituents in production medium. The *Streptomyces* sp. JAJ06 has previously been identified as seawater dependent antibiotic producing actinomycete [5]. Undefined nature of seawater can affect the reproducibility of the production profile of actinomycetes [6]. In this study, the seawater has been expelled from the production medium with incorporation of chemically defined salt formulations. Among the two defined salt formulation, sodium-chloride-based salt formulation favoured maximum antimicrobial compound production in JAJ06. This type of chemically defined salt formulation has already been reported for consistent production of bioactive compounds from seawater dependent *Salinispora tropica* strain NPS21184 [6].

Small manipulations in the culture medium composition can exert significant effect on secondary metabolites biosynthesis in microorganisms [7, 26]. Several researchers working on antibiotics discovery programs have applied PBD and RSM as statistical tools to recognize, manipulate and optimize influencing medium constituents and recorded the increased antibiotic production. For instance, Wang et al., [7] applied RSM approach for medium optimization for antibiotic production by *Xenorhabdus bovienii *and recorded 37.8% increase in antibiotic activity. Recently, Chen et al. [27] reported 2.7-fold increase in antibiotic production by *Bacillus* sp. ZJUIBE-076 using RSM approach.

In the present study, PBD and RSM were applied for medium optimization for antibiotic production by *Streptomyces *sp. JAJ06 and recorded 26.8% increase in antibiotic activity. The results of PBD revealed that the crucial media components related to the antibiotic production by JAJ06 were starch, KBr, and CaCO_3_. Raytapadar and Paul [28] reported starch as a significant media component for production of antibiotic from *Streptomyces aburaviensis* 1DA-28. Similarly, CaCO_3_ has been identified as a crucial ingredients related to the production of cyclic hexapeptide antibiotic by *Streptomyces alboflavus* [29]. RSM was found to be very effective in optimizing the selected medium components evident from positive diagnostic plots (Figure 2) and *R* ^2^ value 0.9831 which was comparable with the earlier reports [7, 27].

## 5. Conclusion

Present investigation focused primarily on improved production of antibiotic by *Streptomyces *sp. JAJ06 as a function of various salt compositions and levels of ingredients in production medium. PBD and RSM were found to be very effective in selecting and optimizing the medium components in manageable number of experimental trials with overall 26.8% increase in antibiotic activity. Moreover, the optimum culture medium obtained in this experiment will be useful for further study with large scale fermentation in a fermenter for the efficient production of antibiotic from this *Streptomyces *sp. JAJ06.

## Figures and Tables

**Table 1:** Low and high levels of each variable used in Plackett-Burman design.

Variables	Medium components	+values (g/L)	−values (g/L)
*X*_1_	Starch	20	2.0
*X*_2_	Yeast extract	8	0.8
*X*_3_	Peptone	4	0.4
*X*_4_	MgSO_4_	2	0.2
*X*_5_	KBr	0.2	0.02
*X*_6_	FeSO_4_·7H_2_O	0.08	0.008
*X*_7_	CaCO_3_	4	0.4

**Table 2:** Plackett-Burman design and experimental response obtained for JAJ06.

Trial	Variables							Antibiotic activity (U/mL) ± SEM
	*X*_1_	*X*_2_	*X*_3_	*X*_4_	*X*_5_	*X*_6_	*X*_7_	*B. subtilis *
1	+	−	+	−	−	−	+	135.0 ± 8.7
2	+	+	−	+	−	−	−	113.3 ± 5.8
3	−	+	+	−	+	−	−	95.0 ± 5
4	+	−	+	+	−	+	−	120.0 ± 0
5	+	+	−	+	+	−	+	146.7 ± 10.4
6	+	+	+	−	+	+	−	133.3 ± 5.8
7	−	+	+	+	−	+	+	120.0 ± 0
8	−	−	+	+	+	−	+	130.0 ± 10
9	−	−	−	+	+	+	−	113.3 ± 5.8
10	+	−	−	−	+	+	+	135.0 ± 8.7
11	−	+	−	−	−	+	+	100.0 ± 5
12	−	−	−	−	−	−	−	95.0 ± 5

**Table 3:** Low, intermediate, and high levels of three variables used in Box-Behnken design for RSM.

Variables	Range and level (g/L)		
	+	0	−
Starch	20	11	2
KBr	0.2	0.11	0.02
CaCO_3_	4	2.2	0.4

**Table 4:** Box-Behnken design matrix along with the experimental and predicted responses (antibiotic activity) of *Streptomyces* sp. JAJ06.

Runs	Variables/coded values			Antibiotic activity (U/mL) ± SEM	
	Starch	KBr	CaCO_3_	Experimental	Predicted
1	−	−	0	110.00 ± 10	108.32
2	+	−	0	140.00 ± 5	136.25
3	−	+	0	120.00 ± 10	123.75
4	+	+	0	148.30 ± 2.9	149.97
5	−	0	−	130.00 ± 10	127.29
6	+	0	−	150.00 ± 10	149.36
7	−	0	+	110.00 ± 0	110.64
8	+	0	+	140.00 ± 10	142.71
9	0	−	−	120.00 ± 0	124.39
10	0	+	−	138.30 ± 7.6	137.26
11	0	−	+	110.00 ± 10	111.04
12	0	+	+	131.70 ± 2.9	127.31
13	0	0	0	163.30 ± 5.8	165.34
14	0	0	0	168.30 ± 2.9	165.34
15	0	0	0	166.70 ± 5.8	165.34
16	0	0	0	166.70 ± 5.8	165.34
17	0	0	0	161.70 ± 7.6	165.34

**Table 5:** Growth and antibiotic activity exerted by *Streptomyces* sp. JAJ06 in production media containing various salt compositions.

Salt composition	Antibiotic activity (U/mL)	Mycelial biomass (g/L)
Sterile seawater	130.0	3.496
Sodium chloride based salt formulation I	136.7	3.393
Sodium sulfate based salt formulation II	126.7	3.013

**Table 6:** Statistical analysis of effects of medium constituents on antibiotic activity as per PBD.

Variables	Medium components	Effect	Standard error	*t*-value	*P* value	Confidence level (%)
*X*_1_	Starch	21.667	2.331	4.65	0.010	99.0
*X*_2_	Yeast extract	−3.333	2.331	−0.72	0.514	48.6
*X*_3_	Peptone	5.000	2.331	1.07	0.344	65.6
*X*_4_	MgSO_4_	8.333	2.331	1.79	0.148	85.2
*X*_5_	KBr	11.667	2.331	2.50	0.067	93.3
*X*_6_	FeSO_4_·7H_2_O	1.100	2.331	0.24	0.825	17.5
*X*_7_	CaCO_3_	16.133	2.331	3.46	0.026	97.4

**Table 7:** Regression analysis (ANOVA) of quadratic polynomial model and significance test.

Source	Sum of squares	df	Mean Square	*F* value	*P* value Prob. > *F*	Significance
Model	6959.86	9	773.32	45.19	<0.0001	Significant
Residual	119.79	7	17.11			
Lack of fit	89.92	3	29.97	4.01	0.1064	Not significant
Pure error	29.87	4	7.47			
Cor. total	7079.65	16				

*R* ^2^ = 0.9831, Adj *R* ^2^ = 0.9613.

**Table 8:** Experimental validation of the combined effect of variables under optimized condition and unoptimized conditions on the antibiotic activity of *Streptomyces* sp. JAJ06.

Variables	Level (g/L)		Antibiotic activity (U/mL)			
	Unoptimized	Optimized	Unoptimized	Optimized		
				Predicted	Experimental	Deviation
Starch	10	7.4	136.7	169.07	173.3	2.50
KBr	0.1	0.048				
CaCO_3_	2	0.8				
